# When Structure Affects Function – The Need for Partial Volume Effect Correction in Functional and Resting State Magnetic Resonance Imaging Studies

**DOI:** 10.1371/journal.pone.0114227

**Published:** 2014-12-02

**Authors:** Juergen Dukart, Alessandro Bertolino

**Affiliations:** 1 F. Hoffmann-La Roche, pRED, Pharma Research and Early Development, NORD DTA, Grenzacherstrasse 124, 4070 Basel, Switzerland; 2 Max Planck Institute for Human Cognitive and Brain Sciences, Leipzig, Germany; 3 Department of Basic Medical Science, Neuroscience and Sense Organs, University of Bari, Bari, Italy; National Scientific and Technical Research Council (CONICET)., Argentina

## Abstract

Both functional and also more recently resting state magnetic resonance imaging have become established tools to investigate functional brain networks. Most studies use these tools to compare different populations without controlling for potential differences in underlying brain structure which might affect the functional measurements of interest. Here, we adapt a simulation approach combined with evaluation of real resting state magnetic resonance imaging data to investigate the potential impact of partial volume effects on established functional and resting state magnetic resonance imaging analyses. We demonstrate that differences in the underlying structure lead to a significant increase in detected functional differences in both types of analyses. Largest increases in functional differences are observed for highest signal-to-noise ratios and when signal with the lowest amount of partial volume effects is compared to any other partial volume effect constellation. In real data, structural information explains about 25% of within-subject variance observed in degree centrality – an established resting state connectivity measurement. Controlling this measurement for structural information can substantially alter correlational maps obtained in group analyses. Our results question current approaches of evaluating these measurements in diseased population with known structural changes without controlling for potential differences in these measurements.

## Introduction

Functional and resting state magnetic resonance imaging (fMRI and rsMRI) have now become established tools to investigate brain function and connectivity. Numerous studies in the past decades have applied fMRI and more recently rsMRI-based measurements to evaluate experience and clinical phenotype-related functional alterations [1]–[21]. These studies have provided a vast heterogeneity of findings which have been attributed to group differences in brain functional activity or connectivity. However, the effect of potential between-group structural differences has not been systematically investigated. These structural differences are likely to give rise to partial volume effects defined as differential relative contribution of grey matter, white matter, or cerebrospinal fluid to the observed voxel- or region-wise signal [22]. In our study, we further assume that the observed voxel-wise signal containing these partial volume effects is a linear combination of the signal from the different tissue types. These effects have been largely ignored by making the implicit assumption that they are controlled for in fMRI and rsMRI analyses. This is because within-subject statistical maps and within-subject correlational maps are respectively computed, and are then used to evaluate between-group differences [23], [24] [25], [26]. Moreover, the signal in typical fMRI and rsMRI analyses might be sufficiently strong to make differences in noise levels across groups negligible, in particular when applying strict correction for multiple comparisons. Therefore, it might be argued that partial volume effects introduced by underlying structural differences, potentially present in fMRI and rsMRI analyses, do not strongly affect the final statistics with these kinds of analyses.

However, this assumption is rather questionable especially because of the relatively low spatial resolution of fMRI and the thickness of the targeted cortical structures, which is in the range of 2–3 millimeters [27]. Thus, even subtle structural between-group differences might lead to differences in the amount of partial volume effects contributing to the signal in the corresponding functional voxels and correspondingly to differences in the observed signal [28]. These limitations are particularly applicable to studies of aging and of neurodegenerative disorders which are generally characterized by grey matter loss. For example, reductions in grey matter volume in a region of interest would inevitably lead to increased contribution of cerebrospinal fluid (CSF) to signal measured in the corresponding region. The increased contribution of CSF would lead to higher noise and correspondingly reduce the within-subject statistical estimates such as beta coefficients. The changes in within-subject statistics due to increased partial volume effects would then transfer to second-level statistics, erroneously suggesting between-group functional differences.

Similarly, rsMRI studies commonly use measurements that are based on within-subject metrics such as correlation coefficients computed between different regions over time. These functional connectivity maps (e.g. Fisher's z-transformed or original) are then used to directly compare the different groups or to extract other more advanced connectivity indices such as the total flow or the clustering coefficient. With the same arguments as above, between-group differences in grey matter volume in a region of interest would lead to greater noise level in this region in the group with lower grey matter volume. Correlation coefficients are well known to be strongly affected by noise. Correspondingly, decreased average correlation strength would be observed in this group in the affected region.

Here we investigate the effects of differential partial volume contribution from grey matter, white matter, and cerebrospinal fluid on results of typical fMRI and rsMRI analyses.

## Methods

### Generated data

**Figure 1:**
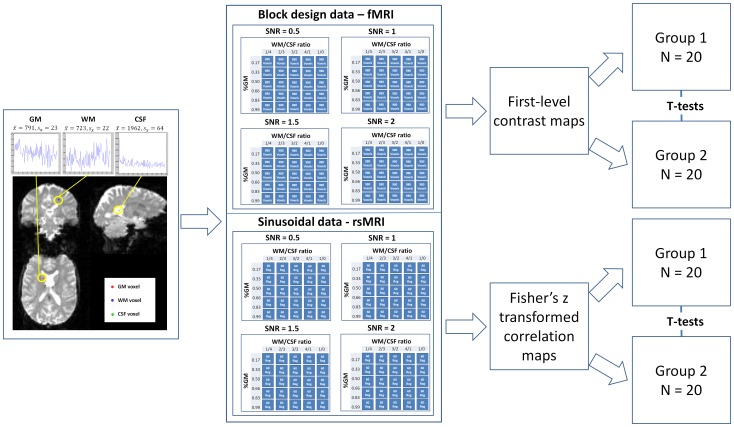
Schematic overview of the data generation procedure and statistical testing performed in this study. *x* – mean of the corresponding voxel time series, *s_x_* – standard deviation of the corresponding voxel time series, GM – grey matter, WM – white matter, CSF – cerebrospinal fluid, fMRI – functional magnetic resonance imaging, SNR – signal-to-noise ratio.

All data generation steps and statistical analyses were implemented in Matlab 7.12 (MathWorks Inc., Sherborn, MA). To obtain realistic parameters for the data generation procedure, a publicly available fMRI dataset (auditory block design experiment, BOLD/EPI images, 2T Siemens, 96 acquisitions, TR = 7s, voxel: 3×3×3 mm^3^, 64 slices, matrix size 64×64) of a single subject commonly used for teaching purposes was downloaded from the statistical parametric mapping website. The imaging data and a more detailed description of the auditory paradigm and the imaging sequence can be found at the following URL: http://www.fil.ion.ucl.ac.uk/spm/data/auditory. These single subject auditory task fMRI data were then used to manually define three voxels of interest, each located in the centre of an easily localized grey matter, white matter, or cerebrospinal fluid anatomical structure (Figure 1) to reduce partial volume effects introduced by the signal from other tissue types. Means and standard deviations of the signal from each voxel over time were computed to obtain distribution characteristics for each tissue class. The means and standard deviations for white matter and cerebrospinal fluid were then used to generate random Gaussian signal for both tissue types with the same mean and standard deviation as observed in the auditory fMRI data.

**Figure 2:**
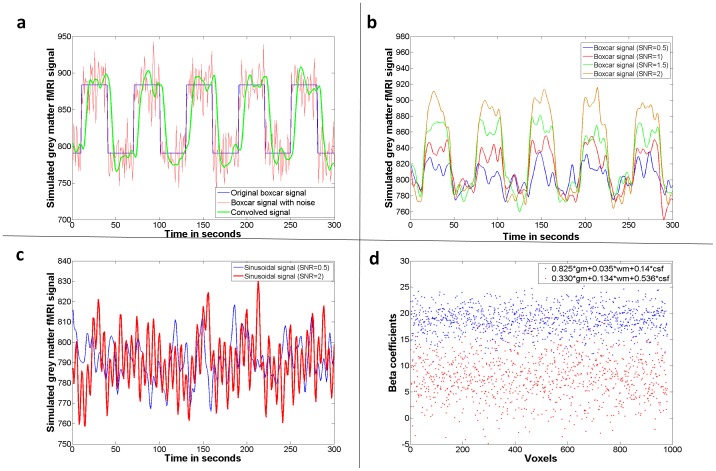
Data generation and statistical results. a) Data generated using the boxcar function to simulate a block design functional magnetic resonance imaging (fMRI) signal. Original signal, signal with Gaussian noise, and the convolved noisy signal are displayed. b) Simulated fMRI time series with four different signal-to-noise ratios are displayed. c) Simulated rsMRI time series with two different signal-to-noise ratios are displayed. d) Two exemplary results of the fMRI simulation study for estimation of beta coefficients are displayed for the 980 functional voxels generated for each constellation of partial volume effect contribution. gm – grey matter, wm – white matter, csf – cerebrospinal fluid, SNR – signal-to-noise ratio.

To generate grey matter signal of a block design fMRI experiment, a boxcar function with the mean corresponding to the mean of the grey matter voxel was computed generating data for 300 time bins and a block duration of 30 seconds (Figure 2a and Figure S1 in File S1). For rsMRI analyses, as these mostly focus on correlation-based measurements, a sinusoidal shape function with the same mean and also 300 time bins was used to generate the voxel-wise grey matter signal. Noise in the grey matter signal was simulated by adding to the boxcar function and to the sinusoidal function random uncorrelated Gaussian noise with the standard deviation corresponding to that observed in the original grey matter voxel. As real fMRI and rsMRI data may contain correlated noise across the different tissue types, e.g. due to subjects' motion, we additionally generated data with correlated (r = 0.2, across tissue types and voxels) random Gaussian noise for both types of analyses. To evaluate how different signal-to-noise ratios affect the results, the grey matter signal using the boxcar- or the sinusoidal function was generated for four different signal-to-noise ratios (0.5, 1, 1.5, and 2) by keeping the noise constant whilst scaling the signal with a factor determined as signal-to-noise ratio multiplied with the standard deviation of noise (Figure 2b,c). The signal for the fMRI simulation study thereby refers to the activation level with respect to the baseline of the simulated block paradigm [29]. For the rsMRI, signal refers to the standard deviation of generated sinusoidal signal without noise. Noise refers in both cases to the standard deviation of random fluctuations added on top of the grey matter signal. The grey matter fMRI and rsMRI signal was convolved with a hemodynamic response function using the spm_get_bf function provided by the SPM8 software package (Statistical Parametric Mapping software: http://www.fil.ion.ucl.ac.uk/spm/) with a time bin length of 1 second.

Further, to simulate partial volume effects, 30 different constellations of the mixture between grey matter, white matter, and cerebrospinal fluid signal were generated (Table 1) using linear combinations of signal from each tissue type. Thereby, for each of the 6 different ratios of white matter and cerebrospinal fluid contribution, 5 different percent contributions of grey matter were generated (Table 1). Following this procedure, 40 datasets were generated separately for fMRI and rsMRI for each of the 4 signal-to-noise ratios and each of the 30 constellations of differential tissue contributions (4*30 = 120 combinations). Each dataset included 980 functional voxels. For rsMRI correlational analyses, only 60 functional voxels per subject were generated for computational reasons. These 40 datasets were then split into two equal groups of 20 each and used for subsequent statistical analyses.

**Table 1:** Differential partial volume effect constellations.

			WM/CSF ratio		
Tissue proportion	1/4	2/3	3/2	4/1	1/0
**GM/WM/CSF**	[0.165,0.167,0.668]	[0.165,0.334,0.501]	[0.165,0.501,0.334]	[0.165,0.668,0.167]	[0.165,0.835,0]
**GM/WM/CSF**	[0.330,0.134,0.536]	[0.330,0.268,0.402]	[0.330,0.402,0.268]	[0.330,0.536,0.134]	[0.330,0.670,0]
**GM/WM/CSF**	[0.495,0.101,0.404]	[0.495,0.202,0.303]	[0.495,0.303,0.202]	[0.495,0.404,0.101]	[0.495,0.505,0]
**GM/WM/CSF**	[0.660,0.068,0.272]	[0.660,0.136,0.204]	[0.660,0.204,0.136]	[0.660,0.272,0.068]	[0.660,0.340,0]
**GM/WM/CSF**	[0.825,0.035,0.140]	[0.825,0.070,0.105]	[0.825,0.105,0.070]	[0.825,0.140,0.035]	[0.825,0.175,0]
**GM/WM/CSF**	[0.990,0.002,0.008]	[0.990,0.004,0.006]	[0.990,0.006,0.004]	[0.990,0.008,0.002]	[0.990,0.010,0]

Different constellations of grey matter (GM), white matter (WM) and cerebrospinal fluid (CSF) contribution simulated in this study to evaluate the impact of partial volume effects on functional and resting state magnetic resonance imaging.

### Real data

To evaluate effects of structural information on rsMRI measurements, a freely available dataset of 21 healthy control subjects (11M/10F, 22–61 years old) comprising rsMRI and T1-weighted data was downloaded from www.nitrc.org. A detailed description of this dataset including sequence parameters is provided in Landman et al. [30]. In brief, for each subject 7 min (210 time points, TR/TE = 2000/30 ms) of rsMRI data were acquired using a 2D echo planar imaging sequence with an in-plane resolution of 3×3 mm (240 mm field of view) and thirty-seven 3 mm transverse slices with 1 mm slice gap. Structural data were obtained using a MPRAGE sequence (TR/TE/TI = 6.7/3.1/842 ms) with a 1×1×1.2 mm^3^ resolution. Only data from the first of two available MRI sessions were used for each subject.

Preprocessing of imaging data was performed using the statistical parametric mapping software (SPM12b, http://www.fil.ion.ucl.ac.uk/spm/software/spm12/) implemented in Matlab 7.12. Preprocessing comprised motion correction of rsMRI data, co-registration to the structural scans, segmentation and spatial normalization of structural data preserving the concentration to MNI (Montreal Neurological Institute) space using unified segmentation [31], application of these spatial normalization parameters to the co-registered functional data, and smoothing by a Gaussian kernel with 8 mm FWHM (full width at half maximum). In the spatial normalization step, the initial resolution of 3×3×3 mm^3^ was kept for rsMRI data to avoid artificial up- or downsampling. Z-transformed voxel-wise degree centrality metrics were extracted from the preprocessed rsMRI images using the REST toolbox [32] with default settings (Removing linear trend: “Detrend” option and “Bandpass” filtering with a high- and low-pass frequency filter of 0.01 and 0.08 Hz). Degree centrality is an established functional connectivity metric representing for each voxel the number of Pearson correlations with all other voxels in the brain exceeding a predefined threshold. The threshold was set to r>.25 as suggested by Buckner et al. [33] for this metric. All computations were restricted to a grey matter mask obtained by thresholding the MNI template used for spatial normalization by a value of 0.2 (>20% probability of being grey matter).

Further, as we wanted to evaluate the relative contribution of grey and white matter tissue to the rsMRI signal in each voxel, the segmented and normalized grey and white matter probability maps were also smoothed with a Gaussian kernel of 8 mm FWHM and downsampled to a resolution of 3×3×3 mm^3^ to match the rsMRI data.

### Statistical analyses of generated data

For fMRI analyses, within-subject first-level beta coefficient maps contrasting the two conditions as generated by the boxcar function were computed using the general linear model design for each possible combination of signal-to-noise ratio and amount of partial volume effects (Figure 2d). These first-level contrast maps were then entered into independent samples t-tests comparing for each signal-to-noise ratio the data having differential grey matter contribution but the same white matter to cerebrospinal fluid ratio. To simulate a realistic whole-brain analysis and assuming 10e6 functional voxels for a typical whole-brain fMRI experiment, a conservative Bonferroni threshold of p<.05 corrected for this number of voxels was applied in all fMRI analyses.

For rsMRI analyses, Pearson correlation maps were computed for each dataset between the generated 60 voxels time series resulting in 1770 ((60*60 – 60)/2) inter-voxel correlation coefficients in the left lower triangle. These correlation coefficients were Fisher's z-transformed to approximate a Gaussian distribution and entered into an independent samples t-test comparing for each signal-to-noise ratio the data coming from each tissue class combination across the two groups. For these analyses, full Bonferroni correction of p<.05 was applied to control for multiple comparisons.

Importantly, the obtained datasets used for the statistical comparisons do not differ in any other parameters besides the amount of partial volume effects introduced into the data. Correspondingly, all differences observed in group comparisons either in fMRI or rsMRI analyses can be attributed to differences in the amount of partial volume effects. Functional measurements are expected to reflect true functional but not structural differences. Accordingly, all significant between-group differences induced by partial volume effects in this study are considered as false positive errors as they do not reflect true functional differences.

### Statistical analyses of real data

In a first analysis, we aimed to evaluate how grey and white matter signal in each voxel contribute to the degree centrality value observed in the corresponding voxel. For this, we deployed a leave-one-out approach to compute voxel-wise general linear models (GLMs) predicting degree centrality values using voxel-wise grey and white matter information. Thereby, voxel-wise GLMs obtained using all but one subjects are used to predict degree centrality of the subject who was not used for training. We then computed correlations between predicted and observed degree centrality maps for each subject.

As we assume that regions with a higher degree of partial volume effects as indicated by their white matter and cerebrospinal fluid contribution show higher noise levels, we would expect these regions to be stronger desynchronized with respect to other brain regions. Correspondingly, we would expect lower degree centrality values for regions with higher contributions from non-grey matter tissues. To test this hypothesis, we divided all voxel-wise degree centrality values obtained for all subjects into 10 chunks, based either on white matter or cerebrospinal fluid probability values (defined as 1 – grey matter – white matter) observed in the corresponding voxels. Each chunk was defined as a white matter or cerebrospinal fluid range of 0.1, e.g. all white matter voxels with values between 0 and 0.1 or between 0.1 and 0.2 and so on. We then computed independent samples t-tests to compare degree centrality values observed in the chunks defined by white matter or cerebrospinal fluid values applying a Bonferroni-corrected threshold of p<.05.

Lastly, we evaluated the impact of partial volume effects on statistical maps obtained using standard SPM regression analyses. For this, we computed in a first step voxel-wise GLMs for all subjects predicting degree centrality values using corresponding grey and white probabilities. In a second step, we then performed two standard SPM regression analyses with age and gender as covariates first using the observed degree centrality values and then second time using residual degree centrality values after removing the variance explained by the GLM computed in step one (from here on referred to as adjustment). As we were not interested in age and gender effects per se but rather in similarity of statistical maps obtained with and without adjustment of degree centrality maps for structural information, a liberal threshold of p<0.05 at voxel level with a cluster threshold of>30 voxels was applied in these analyses. We then evaluated positive and negative correlations with age and gender in both analyses resulting in four statistical maps for each. To assess the similarity between obtained statistical maps, Jaccard indices were computed for the binarized maps obtained with and without adjustment. This index of similarity is defined as the size of the intersection divided by the size of the union of the observed statistical maps. It equals one when a perfect and zero when no overlap exists.

## Results

### Generated data

**Figure 3:**
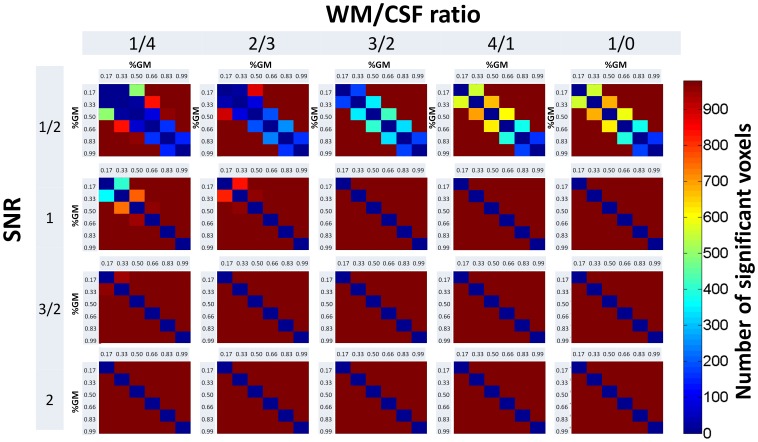
Results of the functional magnetic resonance imaging simulation study. Numbers of significant voxels detected for each signal-to-noise ratio (SNR), grey matter contribution (GM), and white matter (WM) to cerebrospinal fluid ratio (CSF) ratio are displayed as a colour scale. The colour scale indicates the number of significant voxels detected for each partial volume effect constellation (out of 980).

The comparison of fMRI data with differential contribution of grey matter signal to the generated functional voxels and uncorrelated noise resulted in a strongly increased number of false positive errors (Figure 3). These increases were observed for all signal-to-noise ratios and for all constellations of white matter to cerebrospinal fluid ratios. The smallest difference in the amount of partial volume contribution tested in this study results in a false positive detection of differences between the generated fMRI datasets. Most importantly, the number of false positive errors strongly increases with an increased signal-to-noise ratio. We observe the highest number of false positive errors when comparing signal with lowest partial volume effect contribution to any other constellation of partial volume effects tested in this study.

When assuming correlated noise, we observe a strong but less increased number of false positive errors for generated fMRI data across the three tissue types. In contrast to uncorrelated data, an increased amount of false positives is associated with a higher CSF contribution and a lower signal-to noise ratio (Figure S2 in File S1).

**Figure 4:**
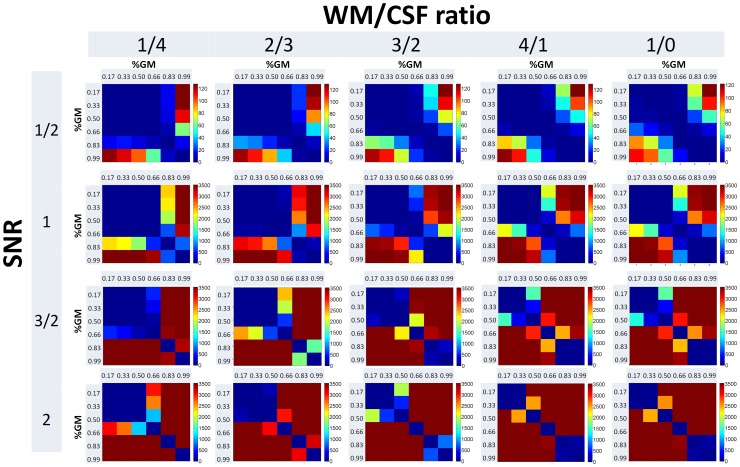
Results of the resting state magnetic resonance imaging simulation study. Numbers of significant voxels detected for each signal-to-noise ratio (SNR), grey matter contribution (GM), and white matter (WM) to cerebrospinal fluid ratio (CSF) ratio are displayed as a colour scale. The colour scale indicates the number of significant connectivity differences detected for each partial volume effect constellation (maximum 3600).

Similarly, in rsMRI analyses a strong dependence is observed between the false positive error rate and the amount of partial volume effects (Figure 4). Though the overall sensitivity of rsMRI analyses to partial volume effects is less evident as compared to fMRI, the amount of partial volume effect- related differences surviving the correction for multiple comparisons is still substantial for most comparisons. Also for rsMRI data, greater signal-to-noise ratio and comparing signal with lowest partial volume effect contribution to other constellations lead to an increased sensitivity to partial volume effects. Higher amounts of false positive errors are thereby observed for combinations with higher grey matter contribution.

For rsMRI data with correlated noise, we find a substantially lower number of false positive errors as compared with correlated data. Higher amounts of false positive errors are associated with higher signal-to-noise ratios and for higher gray matter contribution (Figure S3 in File S1).

### Real data

**Figure 5:**
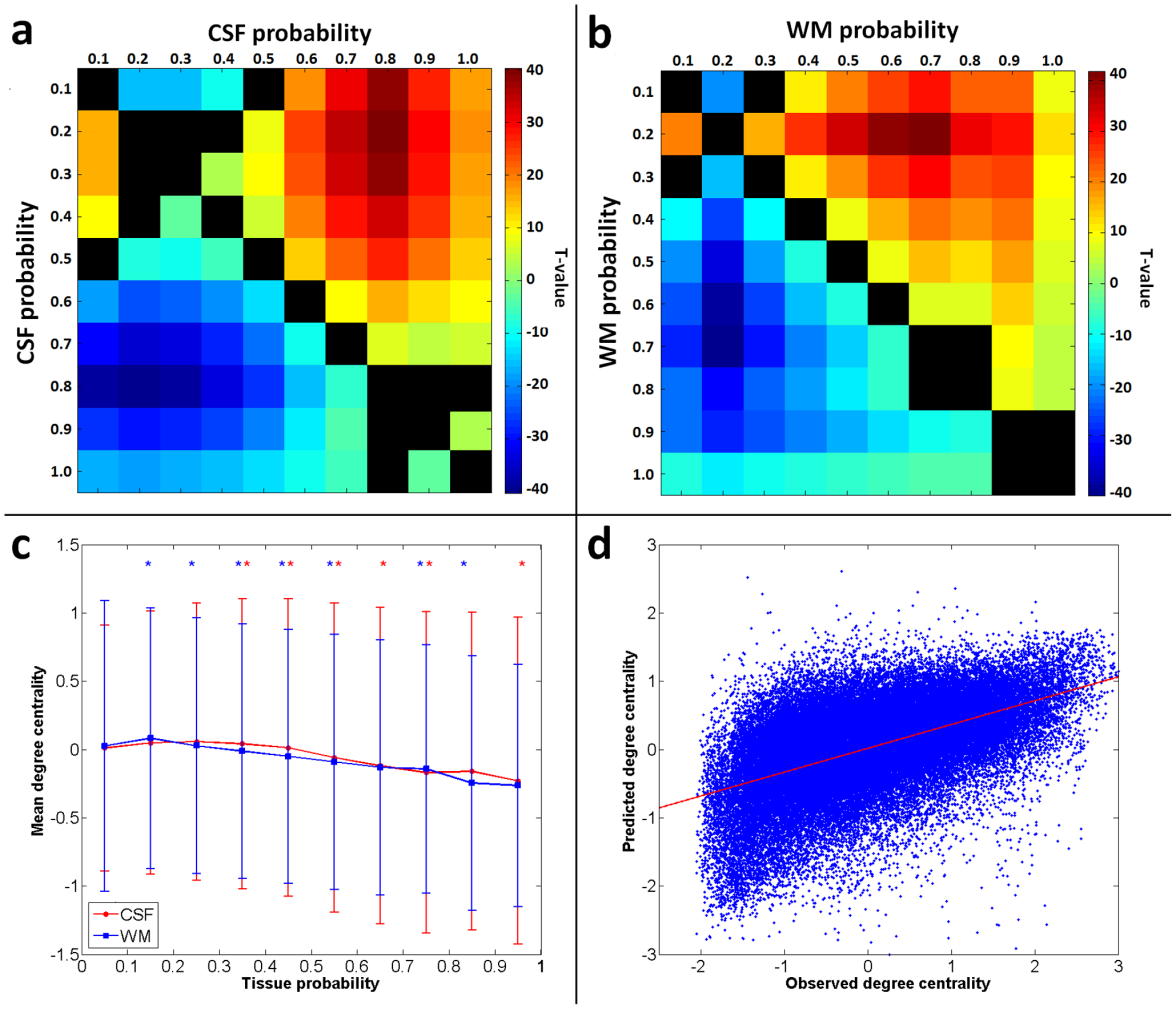
Results of partial volume effects estimation for real resting state magnetic resonance imaging data. a) T-values obtained when comparing voxel-wise degree centrality values grouped by their relative cerebrospinal fluid concentration. Black squares indicate non-signficant results (p<.05 Bonferroni corrected). b) T-values obtained when comparing voxel-wise degree centrality values grouped by their relative white matter concentration. Black squares indicate non-signficant results (p<.05 Bonferroni corrected). c) Mean and standard deviations of degree centralities observed after grouping by their relative cerebrospinal fluid (CSF) or white matter (WM) concentration. *indicates a significantly lower degree centrality value as compared to the next lower contribution of respective tissue. d) A plot of observed vs. predicted degree centrality values for a representative subject in the leave-one-out cross-validation using grey and white matter probabilities to compute the voxel-wise general linear models.

The median correlation strength between observed and predicted degree centrality maps of each subject in the leave-one-out cross-validation was r = .50, corresponding to an explained variance of 25%, with the lowest correlation strength being 0.4 (all p<.001 Bonferroni corrected for multiple comparisons) (Figure 5a).

When comparing degree centrality across different chunks of white matter and cerebrospinal fluid probabilities, significant differences in mean degree centrality are observed for most of the comparisons (Figure 5b–d). Consistently lower degree centrality values are observed in regions with higher white matter or cerebrospinal fluid probabilities.

**Figure 6:**
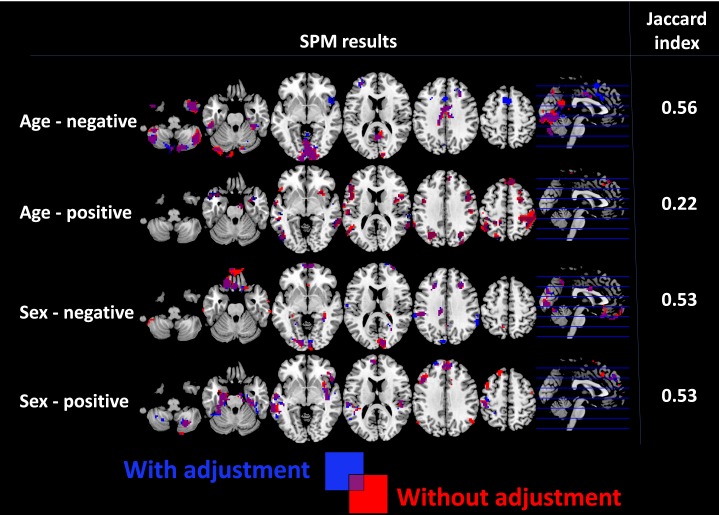
Statistical parametric mapping (SPM) results obtained when testing for negative and positive correlations with age and sex on the group level with and without adjustment for underlying structural information are displayed. On the right, Jaccard indices of overlap for the corresponding statistical maps are shown.

For the four contrasts evaluated in the current study, Jaccard indices between significance maps obtained with and without adjustment of degree centrality values for structural information ranged between 0.22 and 0.56 (Figure 6).

## Discussion

We demonstrate in generated data that small to moderate differences in partial volume effects as induced by differential tissue contribution substantially increase the false-positive rate for both fMRI and rsMRI. These increases are observed for all signal-to-noise ratios evaluated in this study with the highest signal-to-noise ratios being more affected by differences in partial volume effects. Importantly, the generated data used in this study for group comparisons do not differ in any other aspects besides the degree of signal contribution from different tissue types. The observed differences can therefore clearly be attributed to differential partial volume effects. We demonstrate that even minor differences in these can have a strong impact on statistical outcome. We observe the highest number of false-positive errors when groups with a very low degree of partial volume effects are compared to groups with any other partial volume effect contribution evaluated in this study. We further show in a real rsMRI dataset that underlying structural differences are significantly linked to observed functional connectivity measurements and that adjusting for this information can have a substantial impact on the observed group-level statistical findings.

Our findings in real rsMRI data suggest that underlying structural information can explain up to 25% in variance observed in functional connectivity metrics (0.25 determination coefficient, corresponding to the observed median correlation of 0.5 between predicted and observed degree centrality values) with lower degree centrality values observed in regions with higher contributions of non-grey matter tissues. Adjusting for this structural information can have a substantial impact on the observed statistical maps. Importantly, all of these findings refer to a healthy control population. Based on these results and on our results for the generated data, we would expect an even stronger link between structural and rsMRI metrics in diseased populations affected by neurodegenerative processes. Grey matter tissue loss in these populations would lead to a higher contribution of non-grey matter tissues and correspondingly to decreased functional connectivity metrics.

In the simulation part of our study, we further find a lower sensitivity of fMRI and rsMRI data to false positive errors when assuming correlated noise across tissue types and voxels. These findings are not surprising considering that differences in beta coefficients observed between simulated groups with different constellations of partial volume effects are due to differential noise properties across tissue types. Introducing correlations in this noise leads to a more homogeneous combined final signal across the different constellations of grey and white matter contribution and correspondingly to less between group differences in the estimated beta coefficients. Additionally, in rsMRI analyses further differences are introduced due to the fact that correlated noise (across voxels) is recognized as signal when computing correlations. Correspondingly the combined signal becomes more homogenous between simulated groups with different constellations of partial volume effects. Importantly, though the amount of false positive errors due to partial volume effects decreases for both fMRI and rsMRI analyses when assuming correlated noise, these correlations are known to introduce different types of biases as for example repeatedly shown in studies evaluating the effects of motion or scanner instabilities [34]–[36].

The magnitude of structural differences evaluated in this study in generated data is commonly observed in normal aging [37]–[40], in many neurological and psychiatric diseases [38], [41], [42], between males and females [40], [43], but also for learning and treatment-induced structural changes [44]–[47]. Also, the signal-to-noise ratios used in our study for simulation of fMRI and rsMRI data are of comparable amplitude to those evaluated in other simulation studies [29]. If not controlled for, these differences in the underlying structure might significantly bias the interpretation of observed functional and resting state differences. Numerous studies have applied both fMRI and rsMRI without controlling for partial volume effects to study between-group differences in different populations revealing for example significant changes of hippocampal connectivity in Alzheimer's disease [5], [48]. Considering that strong hippocampal atrophy is well validated in this disease, the question remains whether these findings indeed reflect real functional alterations or merely capture the increase in partial volume effects resulting from neurodegeneration of this brain structure. Other studies reported, for example, electroconvulsive therapy induced functional connectivity changes in prefrontal regions in depression [49]. Considering that this treatment is known to induce structural changes, the underlying nature of the observed functional differences would require further exploration [47], [50], [51].

Intriguingly, the number of false positive errors for both fMRI and rsMRI strongly increases with an increased signal-to-noise ratio. This effect is due to the fact that greater signal-to-noise ratio also leads to initially higher average beta and correlation coefficients as compared to a noisier signal. Introducing partial volume effects increases the noise level and leads to a stronger average decrease of the extracted statistical measurements. Correspondingly, the group statistics become more sensitive to differences introduced by the differential degree of partial volume effects. Moreover, fMRI and, in particular, rsMRI studies often apply region-of-interest approaches extracting mean values from predefined anatomical brain areas but without restricting computations of mean values to the grey matter compartments of corresponding regions [52]–[54]. This procedure leads to even stronger averaging of signal from different tissue types and therefore magnifies partial volume effects and correspondingly the risk of false positives. It is important to note that partial volume effects have been for decades a central methodological focus in studies applying positron emission tomography [22], [28], [55]–[58]. These studies have resulted in a large number of tools and methodological developments which allow one to control for partial volume effects using, for example, tissue probability estimates obtained from high resolution structural magnetic resonance scans [22], [55], [56]. Studies applying fMRI and rsMRI as more recent developments have largely ignored these effects and methodological advances by assuming that the applied statistical procedure provides a sufficient control for these effects. As shown in this study, the currently applied fMRI and rsMRI statistical analyses are strongly affected by partial volume effects induced by underlying structural differences. Our findings indicate that these effects should be taken into account in future studies to allow a more functional interpretation of fMRI and rsMRI outcomes.

It is important to note that in the simulation part of our study we make several assumptions on the properties of signal and noise in fMRI and rsMRI data. We assume the observed final voxel-wise signal to be a linear combination of the signal from different tissue types. This assumption is based on geometric properties of the voxel-wise MR signal. It is the most plausible to assume that a tissue covering for example 2/3 of the area covered by a voxel is also contributing 2/3 to the observed signal in the corresponding voxel. In contrast, any other assumption of non-linear contribution would require further assumptions of more complex interactions between tissue types and MR physics, e.g. differential spatial point spread functions for different tissue types. We further assume that noise in grey matter, white matter and cerebrospinal fluid is sufficiently described by a Gaussian distribution. This assumption is also made in commonly applied parametric statistics to analyse fMRI and rsMRI data and has been repeatedly used in previous studies to simulate fMRI data [29], [59]. Lastly, in our study we only evaluate the situation assuming either uncorrelated noise or a correlation of 0.2 between tissue types and voxels. All of these assumptions might affect the observed relationship between partial volume effects and the observed functional differences. Deviations from these assumptions in real data might therefore result in different findings regarding the impact of partial volume effects onto rsMRI and fMRI analyses.

Another important issue is related to the correction procedure proposed in our study and concerns the dissociation of partial volume effects from potentially real functional differences which are induced by differences in the underlying structure. The main assumption behind the proposed correction procedure is that the variability in functional signal is different from the one observed in underlying structure. Correspondingly, in case that these are strongly correlated, the proposed approach is unlikely to dissociate between these two types of effects.

To conclude, although simulation approaches as applied in our study are in general very powerful to uncover mechanisms behind hypothesized effects, they are also limited by the necessity of numerous assumptions which might be true or not. Therefore, they cannot be considered as direct evidence for existence of such effects in real data and require further studies to validate the existence and impact of these effects in real fMRI data. Similarly, further studies are also required to establish the impact of these effects onto fMRI and rsMRI differences observed across different pathological conditions.

## Supporting Information

File S1 Contains Figures S1–S3.
(DOCX)

## References

[1] Di Martino A, Yan C-G, Li Q, Denio E, Castellanos FX, et al (2013) The autism brain imaging data exchange: towards a large-scale evaluation of the intrinsic brain architecture in autism. Mol Psychiatry. 10.1038/mp.2013.78 PMC4162310 23774715

[2] Kamphausen S, Schröder P, Maier S, Bader K, Feige B, et al (2012) Medial prefrontal dysfunction and prolonged amygdala response during instructed fear processing in borderline personality disorder. World J Biol Psychiatry. 10.3109/15622975.2012.665174 22404662

[3] Zhou J, Gennatas ED, Kramer JH, Miller BL, Seeley WW (2012) Predicting regional neurodegeneration from the healthy brain functional connectome. Neuron 73:1216–1227. 22445348 10.1016/j.neuron.2012.03.004 PMC3361461

[4] Ashwin C, Baron-Cohen S, Wheelwright S, O'Riordan M, Bullmore ET (2007) Differential activation of the amygdala and the ‘social brain' during fearful face-processing in Asperger Syndrome. Neuropsychologia 45:2–14. 16806312 10.1016/j.neuropsychologia.2006.04.014

[5] Wang L, Zang Y, He Y, Liang M, Zhang X, et al (2006) Changes in hippocampal connectivity in the early stages of Alzheimer's disease: evidence from resting state fMRI. Neuroimage 31:496–504. 16473024 10.1016/j.neuroimage.2005.12.033

[6] Koshino H, Carpenter PA, Minshew NJ, Cherkassky VL, Keller TA, et al (2005) Functional connectivity in an fMRI working memory task in high-functioning autism. Neuroimage 24:810–821. 15652316 10.1016/j.neuroimage.2004.09.028

[7] Rombouts SA, Barkhof F, Goekoop R, Stam CJ, Scheltens P (2005) Altered resting state networks in mild cognitive impairment and mild Alzheimer's disease: an fMRI study. Human brain mapping 26:231–239. 15954139 10.1002/hbm.20160 PMC6871685

[8] Johnson SC, Saykin AJ, Baxter LC, Flashman LA, Santulli RB, et al (2000) The relationship between fMRI activation and cerebral atrophy: comparison of normal aging and Alzheimer disease. Neuroimage 11:179–187. 10694460 10.1006/nimg.1999.0530

[9] Nyberg L, Eriksson J, Larsson A, Marklund P (2006) Learning by doing versus learning by thinking: an fMRI study of motor and mental training. Neuropsychologia 44:711–717. 16214184 10.1016/j.neuropsychologia.2005.08.006

[10] Rubia K, Overmeyer S, Taylor E, Brammer M, Williams S, et al (2000) Functional frontalisation with age: mapping neurodevelopmental trajectories with fMRI. Neuroscience & Biobehavioral Reviews 24:13–19. 10654655 10.1016/s0149-7634(99)00055-x

[11] Rubia K (2002) The dynamic approach to neurodevelopmental psychiatric disorders: use of fMRI combined with neuropsychology to elucidate the dynamics of psychiatric disorders, exemplified in ADHD and schizophrenia. Behavioural brain research 130:47–56. 11864717 10.1016/s0166-4328(01)00437-5

[12] Uddin LQ, Supekar K, Menon V (2010) Typical and atypical development of functional human brain networks: insights from resting-state FMRI. Frontiers in systems neuroscience 4:21. 20577585 10.3389/fnsys.2010.00021 PMC2889680

[13] Daselaar SM, Fleck MS, Dobbins IG, Madden DJ, Cabeza R (2006) Effects of healthy aging on hippocampal and rhinal memory functions: an event-related fMRI study. Cerebral Cortex 16:1771–1782. 16421332 10.1093/cercor/bhj112 PMC1810232

[14] Sperling R (2007) Functional MRI studies of associative encoding in normal aging, mild cognitive impairment, and Alzheimer's disease. Annals of the New York Academy of Sciences 1097:146–155. 17413017 10.1196/annals.1379.009

[15] Dennis NA, Kim H, Cabeza R (2007) Effects of aging on true and false memory formation: An fMRI study. Neuropsychologia 45:3157–3166. 17716696 10.1016/j.neuropsychologia.2007.07.003

[16] St Jacques P, Dolcos F, Cabeza R (2010) Effects of aging on functional connectivity of the amygdala during negative evaluation: a network analysis of fMRI data. Neurobiology of aging 31:315–327. 18455837 10.1016/j.neurobiolaging.2008.03.012 PMC3541693

[17] Rocca MA, Colombo B, Falini A, Ghezzi A, Martinelli V, et al (2005) Cortical adaptation in patients with MS: a cross-sectional functional MRI study of disease phenotypes. The Lancet Neurology 4:618–626. 16168930 10.1016/S1474-4422(05)70171-X

[18] Kaiser A, Kuenzli E, Zappatore D, Nitsch C (2007) On females' lateral and males' bilateral activation during language production: a fMRI study. International Journal of Psychophysiology 63:192–198. 16797758 10.1016/j.ijpsycho.2006.03.008

[19] Karama S, Lecours AR, Leroux JM, Bourgouin P, Beaudoin G, et al (2002) Areas of brain activation in males and females during viewing of erotic film excerpts. Human brain mapping 16:1–13. 11870922 10.1002/hbm.10014 PMC6871831

[20] Xue G, Chen C, Jin Z, Dong Q (2006) Language experience shapes fusiform activation when processing a logographic artificial language: an fMRI training study. Neuroimage 31:1315–1326. 16644241 10.1016/j.neuroimage.2005.11.055

[21] Koch W, Teipel S, Mueller S, Benninghoff J, Wagner M, et al (2012) Diagnostic power of default mode network resting state fMRI in the detection of Alzheimer's disease. Neurobiology of aging 33:466–478. 20541837 10.1016/j.neurobiolaging.2010.04.013

[22] Muller-Gartner HW, Links JM, Prince JL, Bryan RN, McVeigh E, et al (1992) Measurement of radiotracer concentration in brain gray matter using positron emission tomography: MRI-based correction for partial volume effects. J Cereb Blood Flow Metab 12:571–583. 1618936 10.1038/jcbfm.1992.81

[23] Smith S (2004) Overview of fMRI analysis. British journal of radiology 77:167–175. 10.1259/bjr/33553595 15677358

[24] Friston KJ, Holmes AP, Worsley KJ, Poline JP, Frith CD, et al (1994) Statistical parametric maps in functional imaging: a general linear approach. Human brain mapping 2:189–210.

[25] Lee M, Smyser C, Shimony J (2013) Resting-state fMRI: a review of methods and clinical applications. American Journal of Neuroradiology 34:1866–1872. 22936095 10.3174/ajnr.A3263 PMC4035703

[26] Van Den Heuvel MP, Hulshoff Pol HE (2010) Exploring the brain network: a review on resting-state fMRI functional connectivity. European Neuropsychopharmacology 20:519–534. 20471808 10.1016/j.euroneuro.2010.03.008

[27] von Economo CF, Koskinas GN (1925) Die cytoarchitektonik der hirnrinde des erwachsenen menschen: J. Springer.

[28] Thomas BA, Erlandsson K, Modat M, Thurfjell L, Vandenberghe R, et al (2011) The importance of appropriate partial volume correction for PET quantification in Alzheimer's disease. European journal of nuclear medicine and molecular imaging 38:1104–1119. 21336694 10.1007/s00259-011-1745-9

[29] De Martino F, Valente G, Staeren N, Ashburner J, Goebel R, et al (2008) Combining multivariate voxel selection and support vector machines for mapping and classification of fMRI spatial patterns. Neuroimage 43:44–58. 18672070 10.1016/j.neuroimage.2008.06.037

[30] Landman BA, Huang AJ, Gifford A, Vikram DS, Lim IAL, et al (2011) Multi-parametric neuroimaging reproducibility: A 3-T resource study. Neuroimage 54:2854–2866. 21094686 10.1016/j.neuroimage.2010.11.047 PMC3020263

[31] Ashburner J, Friston KJ (2005) Unified segmentation. Neuroimage 26:839–851. 15955494 10.1016/j.neuroimage.2005.02.018

[32] Song X-W, Dong Z-Y, Long X-Y, Li S-F, Zuo X-N, et al (2011) REST: a toolkit for resting-state functional magnetic resonance imaging data processing. PloS one 6:e25031. 21949842 10.1371/journal.pone.0025031 PMC3176805

[33] Buckner RL, Sepulcre J, Talukdar T, Krienen FM, Liu H, et al (2009) Cortical hubs revealed by intrinsic functional connectivity: mapping, assessment of stability, and relation to Alzheimer's disease. The Journal of Neuroscience 29:1860–1873. 19211893 10.1523/JNEUROSCI.5062-08.2009 PMC2750039

[34] Purdon PL, Weisskoff RM (1998) Effect of temporal autocorrelation due to physiological noise and stimulus paradigm on voxel-level false-positive rates in fMRI. Human brain mapping 6:239–249. 9704263 10.1002/(SICI)1097-0193(1998)6:4<239::AID-HBM4>3.0.CO;2-4 PMC6873371

[35] Power JD, Barnes KA, Snyder AZ, Schlaggar BL, Petersen SE (2012) Spurious but systematic correlations in functional connectivity MRI networks arise from subject motion. Neuroimage 59:2142–2154. 22019881 10.1016/j.neuroimage.2011.10.018 PMC3254728

[36] Smith AM, Lewis BK, Ruttimann UE, Ye FQ, Sinnwell TM, et al (1999) Investigation of low frequency drift in fMRI signal. Neuroimage 9:526–533. 10329292 10.1006/nimg.1999.0435

[37] Good CD, Johnsrude IS, Ashburner J, Henson RN, Friston KJ, et al (2001) A voxel-based morphometric study of ageing in 465 normal adult human brains. Neuroimage 14:21–36. 11525331 10.1006/nimg.2001.0786

[38] Dukart J, Kherif F, Mueller K, Adaszewski S, Schroeter ML, et al (2013) Generative FDG-PET and MRI Model of Aging and Disease Progression in Alzheimer's Disease. PLoS computational biology 9:e1002987. 23592957 10.1371/journal.pcbi.1002987 PMC3616972

[39] Franke K, Ziegler G, Kloppel S, Gaser C (2010) Estimating the age of healthy subjects from T1-weighted MRI scans using kernel methods: exploring the influence of various parameters. Neuroimage 50:883–892. 20070949 10.1016/j.neuroimage.2010.01.005

[40] Smith CD, Chebrolu H, Wekstein DR, Schmitt FA, Markesbery WR (2007) Age and gender effects on human brain anatomy: a voxel-based morphometric study in healthy elderly. Neurobiol Aging 28:1075–1087. 16774798 10.1016/j.neurobiolaging.2006.05.018

[41] Chetelat G, Landeau B, Eustache F, Mezenge F, Viader F, et al (2005) Using voxel-based morphometry to map the structural changes associated with rapid conversion in MCI: a longitudinal MRI study. Neuroimage 27:934–946. 15979341 10.1016/j.neuroimage.2005.05.015

[42] Selvaraj S, Arnone D, Job D, Stanfield A, Farrow TF, et al (2012) Grey matter differences in bipolar disorder: a meta-analysis of voxel-based morphometry studies. Bipolar disorders 14:135–145. 22420589 10.1111/j.1399-5618.2012.01000.x

[43] Good CD, Johnsrude I, Ashburner J, Henson RN, Friston KJ, et al (2001) Cerebral asymmetry and the effects of sex and handedness on brain structure: a voxel-based morphometric analysis of 465 normal adult human brains. Neuroimage 14:685–700. 11506541 10.1006/nimg.2001.0857

[44] Sehm B, Taubert M, Conde V, Weise D, Classen J, et al (2014) Structural brain plasticity in Parkinson's disease induced by balance training. Neurobiology of aging 35:232–239. 23916062 10.1016/j.neurobiolaging.2013.06.021

[45] Gryga M, Taubert M, Dukart J, Vollmann H, Conde V, et al (2012) Bidirectional gray matter changes after complex motor skill learning. Front Syst Neurosci 6:37. 22623914 10.3389/fnsys.2012.00037 PMC3353266

[46] Draganski B, Gaser C, Busch V, Schuierer G, Bogdahn U, et al (2004) Neuroplasticity: changes in grey matter induced by training. Nature 427:311–312. 14737157 10.1038/427311a

[47] Dukart J, Regen F, Kherif F, Colla M, Bajbouj M, et al (2014) Electroconvulsive therapy-induced brain plasticity determines therapeutic outcome in mood disorders. Proceedings of the National Academy of Sciences 111:1156–1161. 10.1073/pnas.1321399111 PMC3903198 24379394

[48] Allen G, Barnard H, McColl R, Hester AL, Fields JA, et al (2007) Reduced hippocampal functional connectivity in Alzheimer disease. Archives of neurology 64:1482–1487. 17923631 10.1001/archneur.64.10.1482

[49] Perrin JS, Merz S, Bennett DM, Currie J, Steele DJ, et al (2012) Electroconvulsive therapy reduces frontal cortical connectivity in severe depressive disorder. Proceedings of the National Academy of Sciences 109:5464–5468. 10.1073/pnas.1117206109 PMC3325678 22431642

[50] Nordanskog P, Dahlstrand U, Larsson MR, Larsson EM, Knutsson L, et al (2010) Increase in hippocampal volume after electroconvulsive therapy in patients with depression: a volumetric magnetic resonance imaging study. J ECT 26:62–67. 20190603 10.1097/YCT.0b013e3181a95da8

[51] Madsen TM, Yeh DD, Valentine GW, Duman RS (2004) Electroconvulsive seizure treatment increases cell proliferation in rat frontal cortex. Neuropsychopharmacology 30:27–34. 10.1038/sj.npp.1300565 15383831

[52] Greicius MD, Supekar K, Menon V, Dougherty RF (2009) Resting-state functional connectivity reflects structural connectivity in the default mode network. Cerebral Cortex 19:72–78. 18403396 10.1093/cercor/bhn059 PMC2605172

[53] Cherkassky VL, Kana RK, Keller TA, Just MA (2006) Functional connectivity in a baseline resting-state network in autism. Neuroreport 17:1687–1690. 17047454 10.1097/01.wnr.0000239956.45448.4c

[54] Zhou Y, Shu N, Liu Y, Song M, Hao Y, et al (2008) Altered resting-state functional connectivity and anatomical connectivity of hippocampus in schizophrenia. Schizophrenia research 100:120–132. 18234476 10.1016/j.schres.2007.11.039

[55] Quarantelli M, Berkouk K, Prinster A, Landeau B, Svarer C, et al (2004) Integrated software for the analysis of brain PET/SPECT studies with partial-volume-effect correction. J Nucl Med 45:192–201. 14960635

[56] Rousset OG, Ma Y, Evans AC (1998) Correction for partial volume effects in PET: principle and validation. J Nucl Med 39:904–911. 9591599

[57] Samuraki M, Matsunari I, Chen WP, Yajima K, Yanase D, et al (2007) Partial volume effect-corrected FDG PET and grey matter volume loss in patients with mild Alzheimer's disease. Eur J Nucl Med Mol Imaging 34:1658–1669. 17520250 10.1007/s00259-007-0454-x

[58] Yanase D, Matsunari I, Yajima K, Chen W, Fujikawa A, et al (2005) Brain FDG PET study of normal aging in Japanese: effect of atrophy correction. Eur J Nucl Med Mol Imaging 32:794–805. 15759148 10.1007/s00259-005-1767-2

[59] Gitelman DR, Penny WD, Ashburner J, Friston KJ (2003) Modeling regional and psychophysiologic interactions in fMRI: the importance of hemodynamic deconvolution. Neuroimage 19:200–207. 12781739 10.1016/s1053-8119(03)00058-2

