# Supplementary material for: When Structure Affects Function – The Need for Partial Volume Effect Correction in Functional and Resting State Magnetic Resonance Imaging Studies
Source: PLoS One. 2014 Dec 2;9(12):e114227. doi: 10.1371/journal.pone.0114227 (PMC4252146; doi:10.1371/journal.pone.0114227)
Supplement: File S1 — Contains Figures S1–S3. (DOCX) [file pone.0114227.s001.docx]

**Supplement 1**


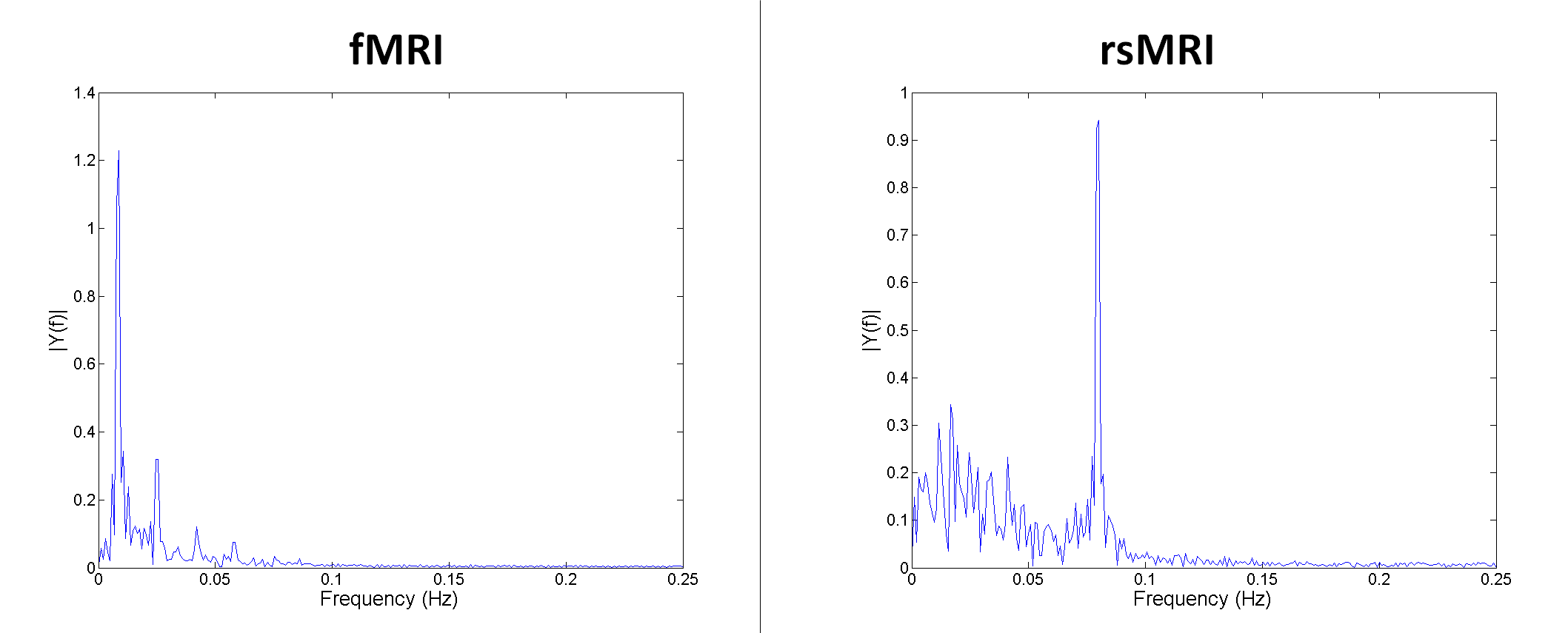


**Figure S1** Absolute amplitude spectrum Y of z-standardized generated voxel-wise time series t for an exemplary fMRI (left) and rsMRI (right) voxel with 99% grey matter contribution (0.2% white matter and 0.8% cerebrospinal fluid).


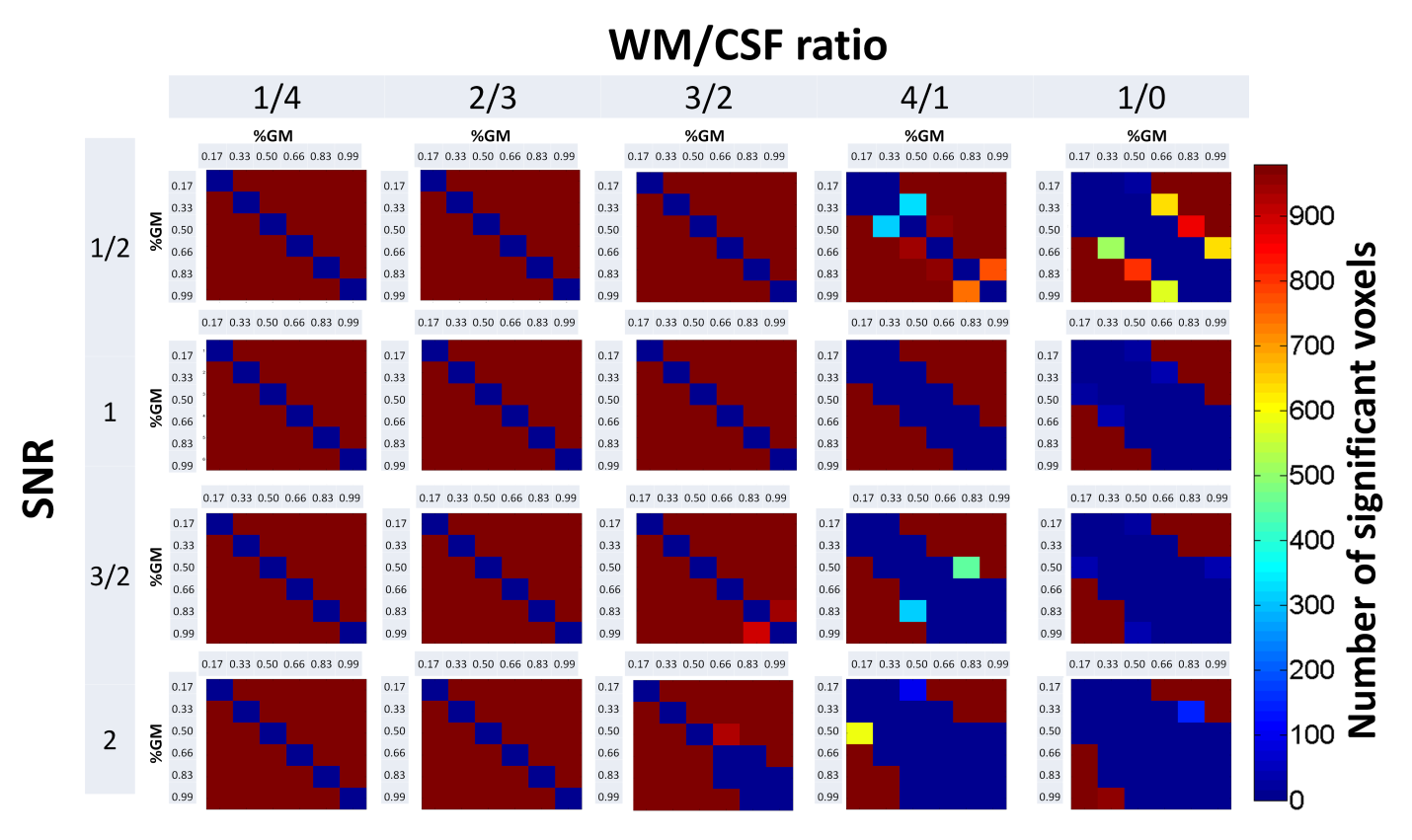


**Figure S2** Results of the functional magnetic resonance imaging simulation study assuming correlated noise (r=0.2). Numbers of significant voxels detected for each signal-to-noise ratio (SNR), grey matter contribution (GM), and white matter (WM) to cerebrospinal fluid ratio (CSF) ratio are displayed as a colour scale. The colour scale indicates the number of significant voxels detected for each partial volume effect constellation (out of 980).


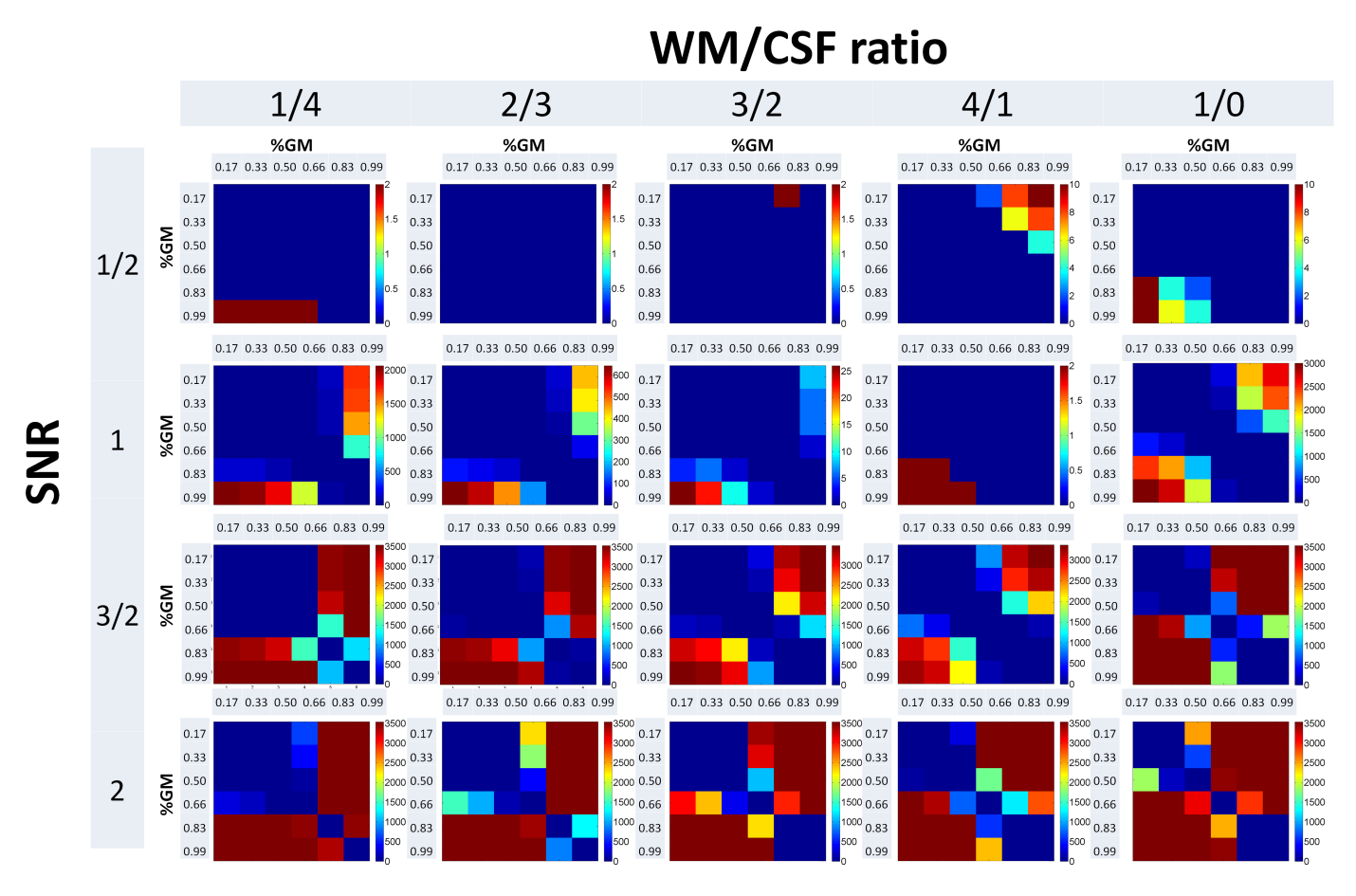


**Figure S3** Results of the resting state magnetic resonance imaging simulation study assuming correlated noise (r=0.2). Numbers of significant voxels detected for each signal-to-noise ratio (SNR), grey matter contribution (GM), and white matter (WM) to cerebrospinal fluid ratio (CSF) ratio are displayed as a colour scale. The colour scale indicates the number of significant connectivity differences detected for each partial volume effect constellation (maximum 3600).
